# Performance Evaluation of Multimodal Multifeature Authentication System Using *K*NN Classification

**DOI:** 10.1155/2015/762341

**Published:** 2015-11-10

**Authors:** Gayathri Rajagopal, Ramamoorthy Palaniswamy

**Affiliations:** ^1^Department of Electronics and Communication Engineering, Sri Venkateswara College of Engineering, Anna University, Sriperumbudur 602117, India; ^2^Department of Electronics and Communication Engineering, Aditya Institute of Technology, Coimbatore 641107, India

## Abstract

This research proposes a multimodal multifeature biometric system for human recognition using two traits, that is, palmprint and iris. The purpose of this research is to analyse integration of multimodal and multifeature biometric system using feature level fusion to achieve better performance. The main aim of the proposed system is to increase the recognition accuracy using feature level fusion. The features at the feature level fusion are raw biometric data which contains rich information when compared to decision and matching score level fusion. Hence information fused at the feature level is expected to obtain improved recognition accuracy. However, information fused at feature level has the problem of curse in dimensionality; here PCA (principal component analysis) is used to diminish the dimensionality of the feature sets as they are high dimensional. The proposed multimodal results were compared with other multimodal and monomodal approaches. Out of these comparisons, the multimodal multifeature palmprint iris fusion offers significant improvements in the accuracy of the suggested multimodal biometric system. The proposed algorithm is tested using created virtual multimodal database using UPOL iris database and PolyU palmprint database.

## 1. Introduction

A multimodal biometric system fuses the evidences presented by multiple biometric traits. Multimodal biometric techniques have received the best recognition because additional information between different biometrics could get improved accuracy. To obtain a successful multibiometric system, one has to essentially implement a good fusing methodology such as match, score, feature, and decision level fusion.

In order to reduce the error rate and to improve the performance accuracy, many researchers worked on multimodal biometric system. Hariprasath and Prabakar [1] proposed a multimodal biometric system using iris and palmprint based on score level fusion and authentication is obtained by hamming distance method. Gargouri Ben Ayed et al. [2] fused fingerprint and faces using match score level fusion using weighted sum method. Here Gabor wavelet network for face and LBP fingerprint features are fused. Abdolahi et al. [3] proposed fuzzy based multimodal biometric system by fusing iris and fingerprint using decision level fusion to provide improved recognition rate. Bahgat et al. [4] fused palm vein and face biometric to obtain the better recognition rate.

Various multimodal score level fusion schemes were proposed by different researchers. Baig et al. [5] proposed score level fusion of iris and fingerprint which is classified using hamming distance calculation. Wang et al. [6] proposed a score level based multimodal biometric combining iris and palmprint using Gaussian mixture model Vatsa et al. [7] combined multi-instant and multiunit iris verification. Wang and Han [8] fused iris and face using score level fusion in which different scores are obtained for different traits and the obtained scores are combined using Support Vector Machine. Wang and Han [9], Kayaoglu et al. [10], Zhang et al. [11], and Peng et al. [12] investigated multimodal biometric fusion using decision and score level fusion.

Monwar and Gavrilova [13] investigated rank level fusion of face, ear, and signature using principal component analysis and Fisher's linear discriminant methods. Kumar and Shekhar [14] investigated multiple palmprint recognition using rank level fusion which uses borda count, bucklin, highest rank, and logistic regression. Match score level fusion using feed forward neural network for the fusion of face and palmprint has been investigated by Thepade and Bhondave [15]. Yang et al. [16] investigated multiple dependency of palmprint using feature level fusion and score level fusion. Wang and Han [9] investigated face iris fusion using score level fusion. Jain et al. [17] investigated the multimodal biometric system based on the face and hand geometry biometrics at the score level.

Conti et al. [18] proposed a multimodal biometric based on two-fingerprint acquisition which uses score level fusion and obtained an improvement of 6% when compared to monomodal biometric based system. Yang et al. [19] used matched score level fusion to fuse hand geometry, fingerprint, and palmprint multimodal biometric system. He used a self-constructed database of 97 subjects. Besbes et al. [20] proposed a hybrid multitrait biometric method using iris and fingerprint. Yang et al. [16] proposed decision level fusion fingerprint templates. Here assessment was taken by individual unimodal assessment through an “AND” operator.

Most significant contribution published in recent years pertaining to multimodal biometric fusion focused extensively on fusing data at the matching score level and decision level. It has been observed that most of the important features are lost on performing data fusion at the latter stages (match score level and decision level). In spite of the abundance of investigations related to multimodal biometrics, relatively little work was done at feature level fusion, since feature fusion has rich information content compared to fusion at the later stages. Therefore, the current exploration on a multimodal biometric fusion at the feature level is anticipated to attain improved recognition accuracy compared to the fusion at the later stages.

## 2. Feature Fusion Using Hierarchical Multiresolution LBP and Gabor

**Figure 1:**
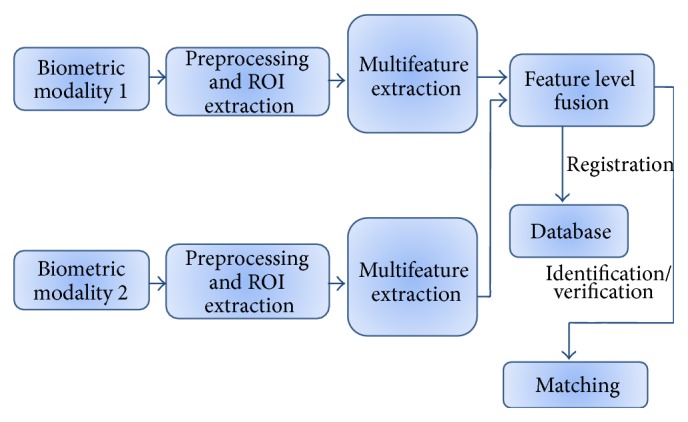
Feature fusion using hierarchical multiresolution LBP and Gabor.

This research mainly discusses the multifeature fusion of palmprint and iris biometrics using feature level fusion. Here, Figure 1 illustrates feature fusion using hierarchical multiresolution LBP and Gabor. It consists of three major blocks preprocessing, feature extraction, and fusion. Multimodal multifeature-based biometric system involves the following steps:

(i) The two modalities presumed are palmprint and iris image which are given as input.
(ii) The Gabor feature and hierarchical multiresolution LBP features of palmprint and iris image, respectively, are taken.
(iii) Images are fused by using feature level fusion.
(iv) *K* nearest neighbor is used for classification.
(v) Recognition accuracy is calculated.

### 2.1. Gabor Wavelets

Gabor wavelets are a filter bank consisting of Gabor filters with diverse scales and rotation. It is efficient for analyzing dissimilar phased features like abrupt ridges or edges. Gabor space is extremely useful in various medical image-processing purposes (Lades et al. [21]). Mainly the Gabor wavelets were developed to represent the receptive fields of simple cells in the visual cortex. However, in practice, they confine to most of the salient properties, together with frequency selectivity, orientation selectivity, and spatial localization. Actually, here the image is conlvolved with a bank of Gabor filters of different orientations and scales. Gabor wavelet has the following general form as in

(1) ψμ1,ν1z=Kμ1,ν12σ2e−Kμ1,ν12Z2/2σ2ejKμ1,ν1Z−e−σ2/2,

where ‖ ‖ represents the norm operator, *ν*_1_ and *μ*_1_ are scale and the orientation, respectively, of the Gabor kernel, *z* = (*x*_1_, *y*_1_) represents a variable in spatial domain, and *K*_*μ*_1_,*ν*_1__ represents wave vector and *σ* is the standard deviation.

**Figure 2:**
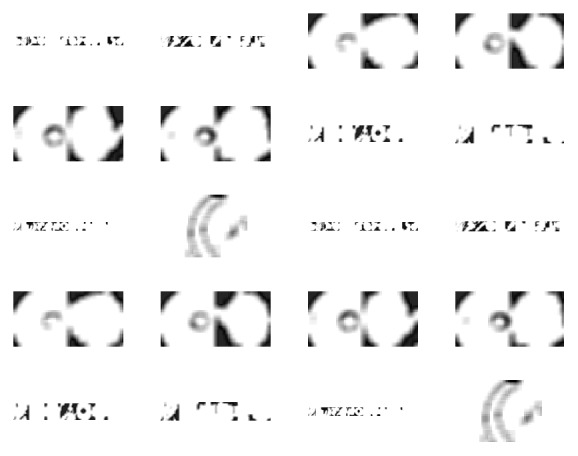
Gabor Kernal Filer Output.

The wave vector is represented in

(2) $$\begin{array}{c} K_{\mu_{1},\nu_{1}}=K_{\nu_{1}}\left(\;\cos\phi_{\mu_{1}}+j\sin\phi_{\mu_{1}}\;\right), \end{array}$$

where *K*_*ν*_1__ = *K*_max_/*f*_*ν*_1__ and *ϕ*_*μ*_1__ = *μπ*/8 with *K*_max_ being the maximum frequency and *f* is the spacing factor. In this research, the Gabor kernel filter used is of three different scales and four orientations.  Figure 2 shows the Gabor kernel filter output.

### 2.2. Hierarchical Multiresolution Local Binary Pattern

Ojala et al. [22] introduced local binary pattern in 1996. The local binary pattern is a gray scale invariant texture measure and is a helpful tool to model texture images. It tags the pixels representation by using threshold of the pixels of the local neighbor around each pixel and considers the result as binary numbers. It is a combining approach to divergent statistical and structural forms of texture analysis. The major property of hierarchical multiresolution LBP is its robustness to monotonic gray scale alterations caused. An added advantage of hierarchical multiresolution LBP is its computational simplicity to analyze images in real time. Hierarchical multiresolution LBP is operated with eight neighbors of a pixel, with the value of the middle pixel as a threshold. Hierarchical multiresolution LBP codes for a neighbor are produced by multiplying the threshold assessment with weights specified to the resultant pixels and the results are summated. It is executed by an orthogonal measure of local contrast. The averages of gray levels under the middle pixel are deduced from that of the gray levels over the center pixel. Two-dimensional distributions hierarchical multiresolution LBP and local contrast measures are used as features.

Local binary pattern (Ojala et al. [22]) is used to capture the local structure of the image. Center pixel of the image is assumed to be *K*_*c*_ = (*x*, *y*) with 8 neighboring pixels (*M* = 8) and radius of the neighborhood is assumed as *r* = 1. The hierarchical multiresolution LBP is obtained as given in

(3) LBPM,rKc=∑mM−1fKn,Kc2n,fKn,Kc=1,0,IKn≥IKc,IKn>IKc,

where *I*(*K*_*n*_) and *I*(*K*_*n*_) are the gray values of the center pixel *K*_*c*_ = (*x*, *y*).

Gray values of *M* neighbouring pixels are obtained using bilinear interpolation and the coordinate of *K*_*n*_ is determined by

(4) $$\begin{array}{c} \left(\;x_{n},y_{n}\;\right)=\left[\;x_{c}+r\cos\left(\;\frac{2\pi n}{M}\;\right),y_{c}-r\sin\left(\;\frac{2\pi n}{M}\;\right)\;\right]. \end{array}$$

To enhance the performance of LBP operator, multiresolution LBP features are used. Multiresolution LBP features consist of richer information than the single LBP operator. Conventionally, LBP features with different scales are obtained and concatenated into a lengthy feature. The obtained feature contains enormous information but it has a drawback of curse of dimensionality.

**Figure 3:**
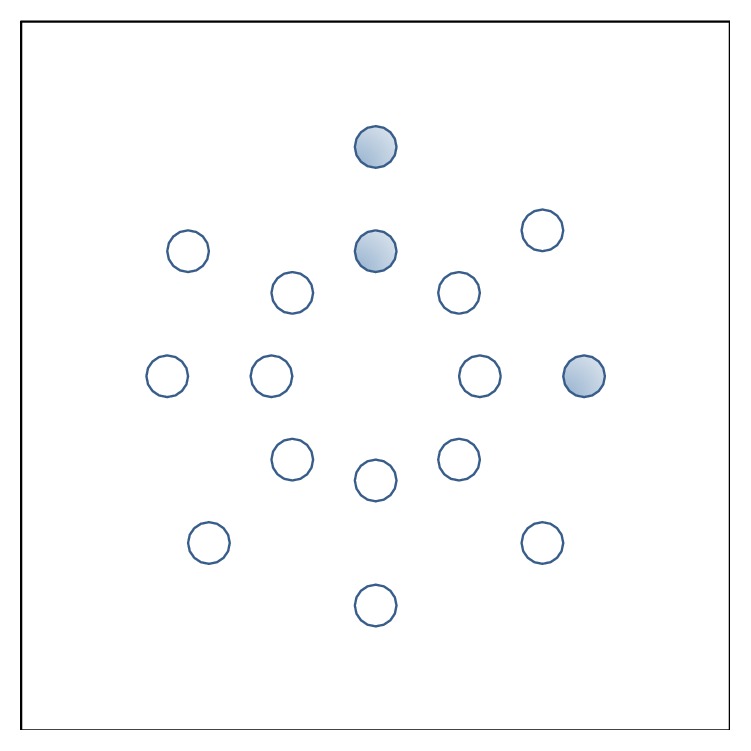
Binary pattern of different radius. Filled pattern represents 1 while the blank circle represents 0.

**Figure 4:**
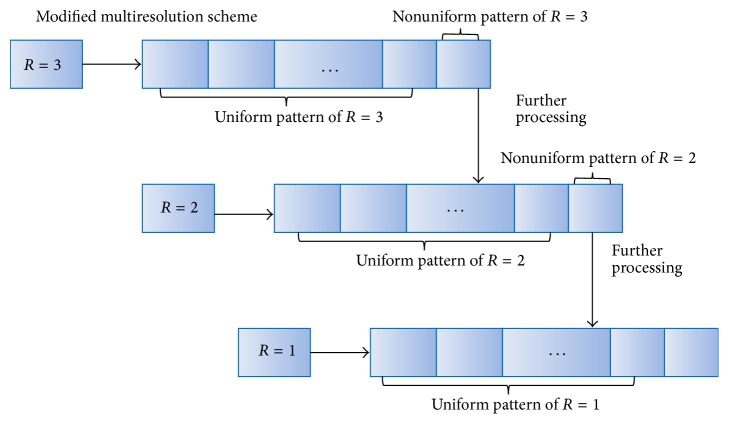
An illustration of proposed hierarchical multiresolution system.

Nonuniform pattern contains more useful information; some of the processing steps are investigated by Raja and Gong [23] and Liao et al. [24]. However, the recognition accuracy depends on the training samples.  Figure 3 explains an illustration of the binary pattern. It consists of nonuniform (bigger radius) and uniform (smaller radius) patterns. For the uniform pattern a subhistogram is constructed, but, for the nonuniform pattern, they are processed to dig out their LBP pattern by smaller dimension. Thus, the processing steps are continued until the pixels patterns are uniform.  Figure 4 explains the proposed multiresolution hierarchical LBP system. Initially, LBP histogram is constructed. Using nonuniform pattern for *R* = 3, a new histogram pattern of *R* = 2 is constructed. Then using nonuniform pattern, *R* = 2 are processed to obtain the histogram pattern of *R* = 1.

## 3. Proposed Multimodal Feature Fusion Block Diagram

**Figure 5:**
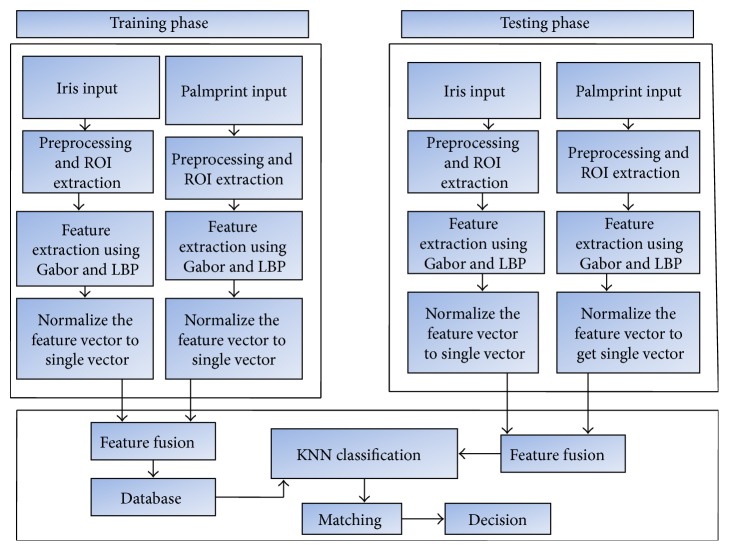
Proposed feature fusion multimodal biometric system.

The proposed methodology for investigating the multimodal multifeature biometric systems is based on the combination of palmprint and iris. Feature fusion has the advantage of exploiting rich information from each biometric.  Figure 5 represents proposed feature fusion multimodal biometric system based on Gabor and hierarchical multiresolution LBP extraction. The feature vectors are extracted independently from the preprocessed images of palmprint and iris. These features are normalized to obtain a single vector. The feature vectors of input images (test image) are then evaluated with the templates of the database (train image) to produce the output. Fusing more than one modality improves the recognition accuracy reduces False Acceptance Rate and False Rejection Rate. The proposed multimodal, multifeature biometric method overcomes the restrictions of single biometric systems and convenes the accuracy requirements.

**Figure 6:**
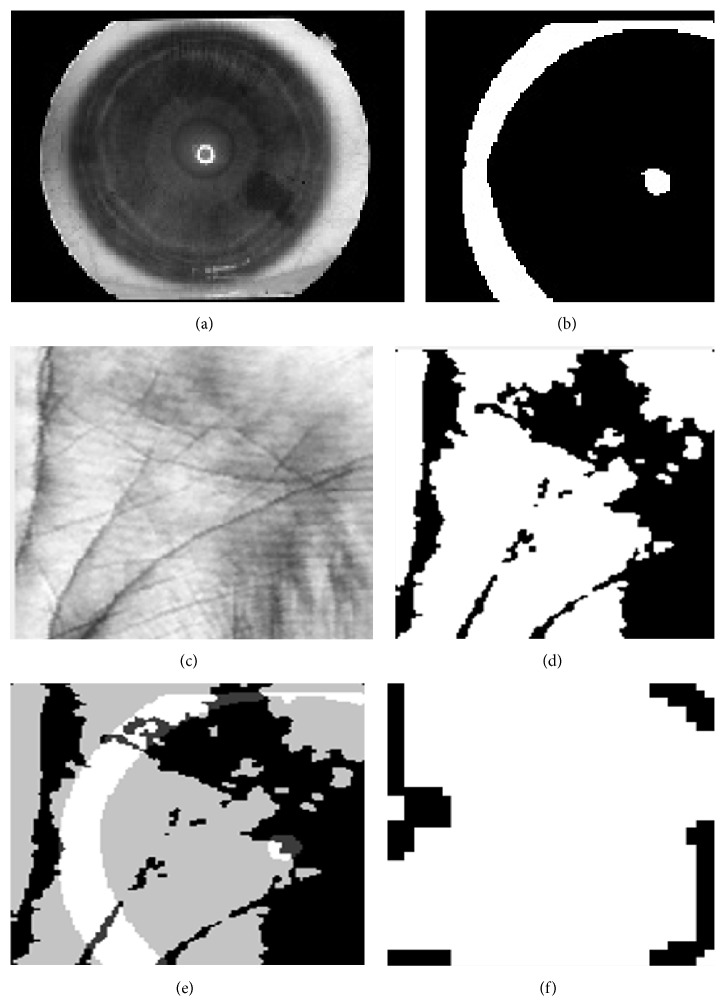
Feature fused image: (a) eye image, (b) preprocessed eye image, (c) palmprint image, (d) preprocessed palmprint image, (e) feature fused image, and (f) segmented image.

**Figure 7:**
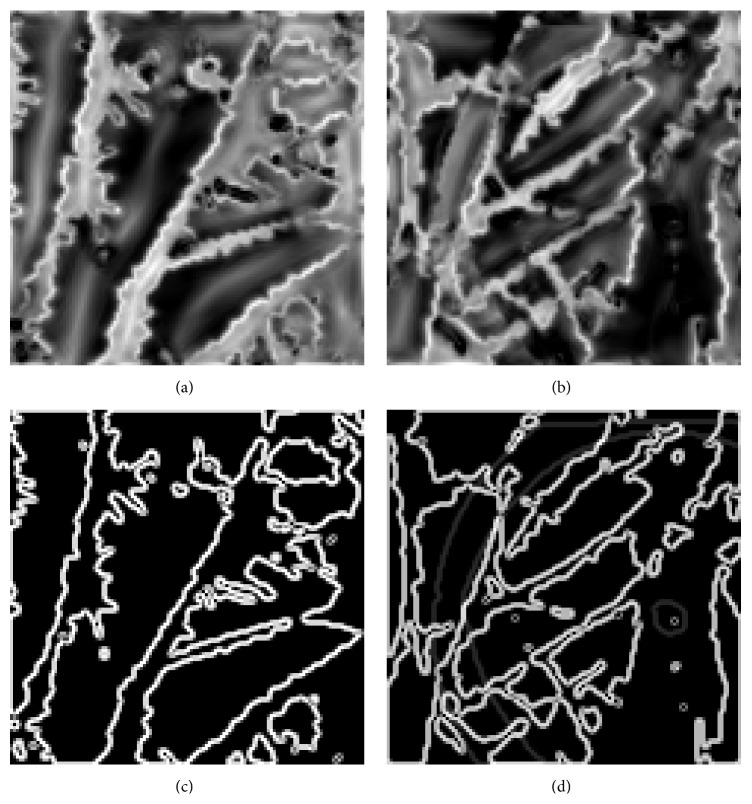
Phase congruency of (a) test image and (b) matched image. Gradient magnitude of (c) test image and (d) matched image.

**Figure 8:**
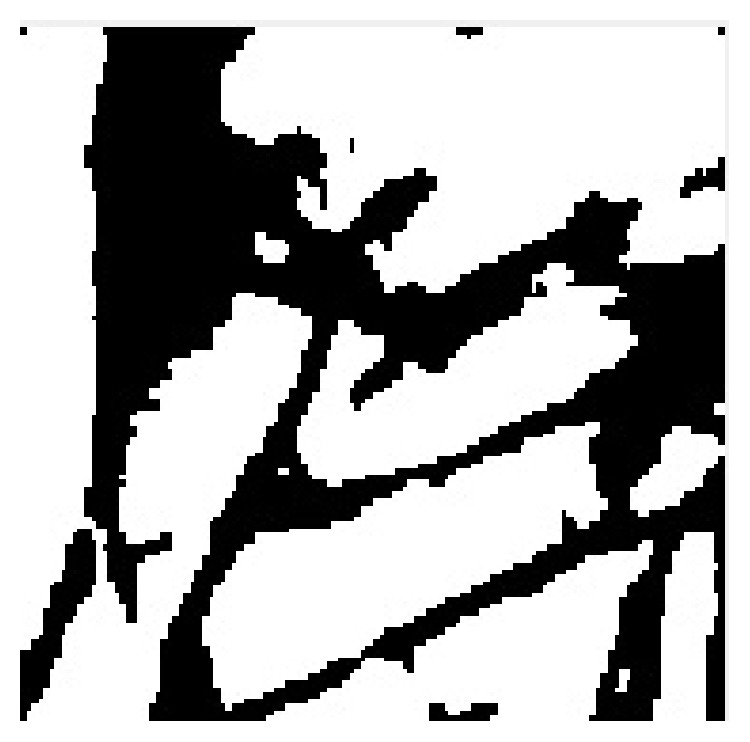
Sample image found during matching.

Figure 6 explains the original image of the iris and palmprint taken from UPOL and PolyU palmprint database. Here, various stages of palmprint and iris image processing are explained, that is, preprocessing of palmprint and iris image, feature level fused image, and segmentation result of the fused image. The proposed multimodal biometric technique exploits most of the information from each monomodal biometric. Gabor and hierarchical multiresolution LBP features are extracted for each palmprint and iris image, and the acquired features are fused by using feature fusion and stored in a database for matching.  Figure 7 illustrates the phase congruency and gradient magnitude extracted from test image, and the matched image is stored in a database.  Figure 8 illustrates a sample image found in the database during matching.

## 4. Result and Discussion

To evaluate the effectiveness of the proposed multimodal biometric system, a database containing palmprint and iris samples are required. To build the virtual multimodal database, images are adopted from PolyU Palmprint database. It includes 7752 images corresponding to 386 subjects. Iris image databases are adopted from UPOL database. It includes 768 images of 576 × 768 pixels captured from 128 subjects in two distinct sessions. Later, each sample of the iris database is randomly merged with one sample of the palmprint database.

**Figure 9:**
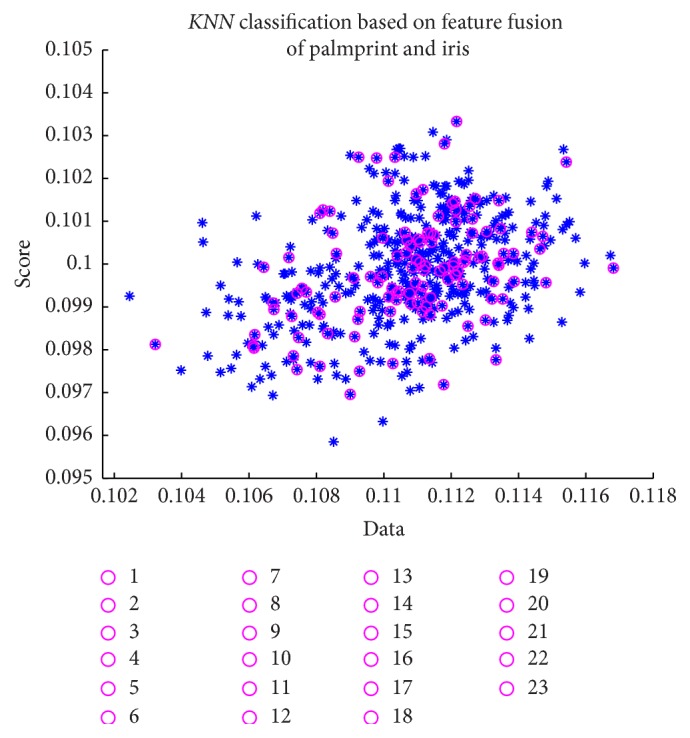
*K*NN classification for the proposed multifeature fusion multimodal biometric.

For the research work, 123 individual palmprint images and iris images are selected; every person has 5 samples and totaling up to 615. Each person's palmprint and iris images were taken as a template (totaling 123). The remaining 492 were used as training samples. The experiments were performed in MATLAB, with image processing Toolbox, on a device with an Intel core 2 Duo CPU processor. Here, among 123 dissimilar test database, untrained images experience similar algorithm as trained image and compare to the original trained image.  Figure 9 explains the *K* nearest neighbor classification result of the proposed multimodal biometric fusion of palmprint and iris. Here, legends with “*o*” of different colours represent the test data of 123 individuals. Symbol “*∗*” represents 492 trained samples of 123 individuals. *K*NN classification is obtained, based on the multifeature fusion (Gabor and hierarchical multiresolution LBP) value of the test and trained image. The proposed multifeature fusion method based on hierarchical multiresolution LBP and Gabor fusing iris and palmprint system achieves a recognition accuracy of 99.98%, with equal error rate (ERR) of 0.0378%.

Twenty samples were taken and analyzed using *K* means algorithm. Sixteen samples were analyzed using *K* nearest neighbor classification algorithm.  Table 1 represents two class ids, assumed for *K* means algorithm. Here, each class id was assumed to have ten classes.  Table 2 represents the matching accuracy obtained for each sample, using *K* means classification algorithm. Here, 20 samples S1 to S20 were considered.  Table 3 represents the class id assumed for *K* nearest neighborhood classification. Here, each class id was assumed to have four classes.  Table 4 represents the matching scores obtained by using *K* nearest neighborh algorithm.

Here, class id one and class id four were matched perfectly because they both belong to same class. It was found that *K*-nearest neighbor algorithm obtained a higher matching accuracy than the *K* means algorithm.

**Figure 10:**
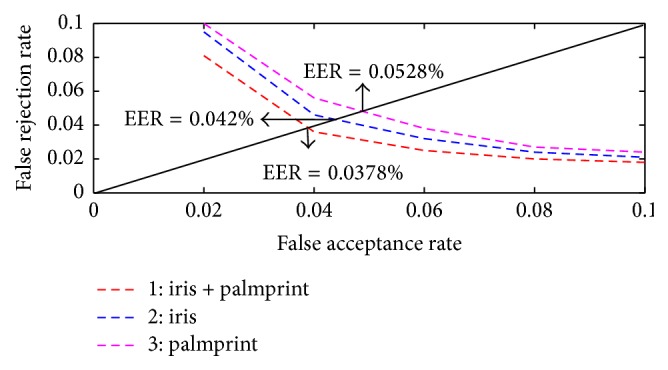
ROC curves for the unimodal and multimodal system.

Figure 10 shows the receiver operating charachteristics (ROC) curve for the unimodal and bimodal biometric system. From the graph it has been observed that the proposed multimodal biometric system acheives a reduced equal error rate (EER) of 0.0378%.

Table 5 explains the comparison of different modality combinations and their recognition accuracy. From the classified result, it was concluded that the performance of the proposed iris palmprint features fusion obtains better recognition accuracy when compared to other fusion methods. Here feature fusion offers enhanced performance compared to other level of fusion. Moreover multifeature (hierarchical multiresolution LBP and Gabor) multimodal (palmprint and iris) feature fusion increases the recognition accuracy. The combination of palmprint and iris (multimodal multifeature fusion) is classified using *K* nearest neighbor; here the distance between test and trained vectors is small when compared to the other combinations discussed so far.

## 5. Conclusion

This research has presented a feature level fusion of multimodal multifeature palmprint and iris recognition system. Gabor wavelets and hierarchical multiresolution LBP are used for feature extraction, and PCA was applied to reduce the dimensionality. Finally, the feature vectors are classified using *K*NN. The experiment result of the proposed multifeature fusion method based on multiresolution hierarchical multiresolution LBP and Gabor fusing iris and palmprint system achieves a recognition accuracy of 99.96%, with equal error rate of 0.0378%, on the publicly available PolyU palmprint and UPOL iris database. Here feature fusion offers enhanced performance compared to other levels of fusion. Moreover, multifeature (hierarchical multiresolution LBP and Gabor) multimodal (palmprint and Iris) feature fusion increases the recognition accuracy. The combination of palmprint and iris (multimodal multifeature fusion) is classified using *K* nearest neighbor; here the distance between test and trained vectors is small when compared to other combinations discussed so far.

## Figures and Tables

**Table 1:** Assumed class id for *K*-means algorithm.

3	2	4	4	3	1	4	4	3	3	Class id 1

1	1	2	2	3	1	5	4	5	5	Class id 2

**Table 2:** Matching scores using *K*-means algorithm.

S1	S2	S3	S4	S5	S6	S7	S8	S9	S10
74.9	72.81	76.99	72.21	0	0	0	0	0	0
78.9	73.51	72.39	71.88	0	0	0	0	0	0
0	0	0	0	88.42	85.78	73.43	87.71	0	0
0	0	0	0	87.76	86.11	66.84	88.97	0	0
0	0	0	0	0	0	0	0	71.63	72.34
74.2	69.21	73.92	77.82	0	0	0	0	0	0
0	0	0	0	0	0	0	0	0	0
0	0	0	0	0	0	0	0	0	0
0	0	0	0	0	0	0	0	0	0
0	0	0	0	0	0	0	0	0	0

S11	S12	S13	S14	S15	S16	S17	S18	S19	S20

0	0	0	0	0	0	0	0	0	0
0	0	0	0	0	0	0	0	0	0
0	0	0	0	0	0	0	0	0	0
0	0	0	0	0	0	0	0	0	0
75.46	74.18	0	0	0	0	0	0	0	0
0	0	0	0	0	0	0	0	0	0
0	0	0	0	0	0	73.79	86.44	70.02	76.72
0	0	69.20	76.20	88.65	86.29	0	0	0	0
0	0	0	0	0	0	67.71	88.85	73.72	76.09
0	0	0	0	0	0	68.19	85.23	69.02	69.76

**Table 3:** Assumed class id for *K*-nearest neighbor algorithm.

1	1	1	4	Class id 1

1	2	3	4	Class id 2

**Table 4:** Matching scores using *K*-nearest neighbor algorithm.

S1	S2	S3	S4	S5	S6	S7	S8
99.18	82.34	81.30	98.45	0	0	0	0
0	0	0	0	98.43	88.76	86.83	99.26
0	0	0	0	0	0	0	0
0	0	0	0	0	0	0	0

S9	S10	S11	S12	S13	S14	S15	S16

0	0	0	0	0	0	0	0
0	0	0	0	0	0	0	0
96.34	85.43	83.34	98.43	0	0	0	0
0	0	0	0	93.34	85.43	86.46	94.89

**Table 5:** Comparison of various modalities.

Method	Recognition accuracy	Modalities
Feature fusion of single scale LBP Guo et al. [25]	81.46	Face and palmprint

Score level fusion Zhou and Bhanu [26]	93.30	Side face and gait

Feature fusion of multiresolution LBP Guo et al. [25]	94.79	Face and palmprint

Score level fusion Kumar et al. [27]	94.59	Hand geometry and palmprint

Score level fusion Nandakumar et al. [28]	94.80	Fingerprint and iris

Feature Fusion of modified multiresolution Guo et al. [25]	96.67	Face and palmprint

Score level fusion Zhang et al. [29]	92.67	Fingerprint and palmprint

Score level fusion Korves et al. [30]	97.50	Fingerprint and face

Decision level fusion Abdolahi et al. [3]	98.20	Fingerprint and iris

Feature fusion Zhou and Bhanu [26]	97.40	Side face and gait

Score level fusion Aguilar et al. [31]	98.20	Iris and palmprint

Rank level fusion Monwar and Gavrilova [13]	98.82	Face, ear, and signature

Proposed feature fusion of hierarchical multiresolution LBP and Gabor	99.96	Iris and palmprint
